# Urban Atmospheric Environment Quality Assessment by Naturally Growing Bryophytes in Central China

**DOI:** 10.3390/ijerph17124537

**Published:** 2020-06-24

**Authors:** Yanbin Jiang, Xifeng Zhang, Ronggui Hu, Jinsong Zhao, Miao Fan, Muhammad Shaaban, Yupeng Wu

**Affiliations:** 1 Key Laboratory of Arable Land Conservation (Middle and Lower Reaches of Yangtze River), Ministry of Agriculture, College of Resources and Environment, Huazhong Agricultural University, Wuhan 430070, China; jiangyanbin@mail.hzau.edu.cn (Y.J.); rghu@mail.hzau.edu.cn (R.H.); jszhao@mail.hzau.edu.cn (J.Z.); fanmiao@webmail.hzau.edu.cn (M.F.); 2 College of Geography and Environmental Science, Northwest Normal University, Lanzhou 730070, China; zhangxifeng@nwnu.edu.cn; 3 Department of Soil Science, Faculty of Agricultural Sciences and Technology, Bahauddin Zakariya University, Multan 60080, Pakistan; shabanbzu@hotmail.com

**Keywords:** air quality, biomonitor, bryophyte diversity, ecological risk, trace elements

## Abstract

Bryophytes are popular biomonitoring plants for atmospheric environments. The objectives of this study were to examine the characteristics of bryophyte communities, determine a suitable monitor species, and assess urban atmospheric environment quality by the joint use of bryophyte features and chemical properties in a large city in China. A pleurocarpous feather moss *Haplocladium angustifolium* was recognized as a good biomonitor of atmospheric deposition in central China by investigating bryophyte communities and habitat environment in various ecological function regions of the urban areas in Wuhan. The concentrations of trace elements, including As, Cd, Co, Cr, Cu, Mn, Ni, V, Pb, and Zn, in moss and soil samples from 25 sampling sites were analyzed by inductively coupled plasma mass spectrometry. The concentrations of Cd and Zn in *Haplocladium angustifolium* collected from the entire study area were much higher than those in substrate soil. Cd was at the highest ecological risk level among the 10 elements, which contributed 34.5% to the potential ecological risk index (RI). An RI value of 392.8 indicated that urban atmospheric quality in Wuhan was in a considerable potential ecological risk. The index of atmospheric purity, regarding species richness, cover, and frequency of bryophytes, was spatially and negatively correlated with RI, also demonstrated the atmospheric quality. Effective measures should be considered to alleviate certain airborne trace element contamination and protect the environment and human health in this metropolis.

## 1. Introduction

Atmospheric pollution due to population growth and shift, the increases in urbanization and industrialization, and the continuous increase of motorized transportation is one of the major problems in urban areas and is a source of great public concern regarding environmental and health consequences [1,2,3]. Various approaches and models for environmental assessment, particularly air quality assessment, such as sampling of bulk, dry, or wet deposition, and the measurement of living organisms including insects, birds, cryptogams (bryophytes and lichens), angiospermous leaves and barks, or gymnospermous needles, have been applied [4,5]. Bryophytes are popular indicator/monitor plants because they cause fewer technical and analytical problems than lichens or tree bark [6,7,8] and can be considered to be complementary to conventional bulk deposition analysis for addressing temporal and spatial patterns [9]. The investigation of naturally growing bryophytes in specific regions is appropriate for extensive monitoring studies [10,11] and for understanding the responses of bryophytes to atmospheric environmental heterogeneity [12]. However, atmospheric deposition of trace elements cannot be accurately estimated from the concentrations of trace elements in bryophyte tissues [9], and it is impossible to isolate their effects from those of other environmental variables [13].

When using naturally growing bryophytes as biomonitors, either the composition and structure of bryophyte communities (species richness, cover, and life forms) and the development of certain bryophytes is considered [14], or certain depositions, which are mainly trace elements, such as nitrogen and sulfur, are measured in such native moss species as *Hypnum cupressiforme*, *Haplocladium microphyllum*, *Pleurozium schreberi*, and *Pseudocleropodium purum* [6,15,16]. The types of moss species which could be selected for bioindication/biomonitoring and assessment of the environment of a certain area depend on not only their morphological and physicochemical characteristics, but also on their occurrence and abundance in the study region [17,18]. The structural and ecological characteristics responsible for the adaptation of bryophytes to an urban environment with open habitat and the existence of pollution are their high capacity for vegetative propagation and growth forms with short turfs, cushions, or mats [19,20].

Biomonitoring techniques by bryophytes have been widely employed worldwide, especially in Europe [21,22]. In China, mosses have been used as biomonitors to assess atmospheric quality in several studies and cities [23,24,25]. However, several cities in the process of rapid urbanization, such as Wuhan, have not been included. Considering the drawbacks of chemical analysis of atmospheric deposition, the joint use of bryophyte features and chemical properties via the index of atmospheric purity (IAP) method and accumulation of airborne pollutants, which has been applied only in few studies, was employed to evaluate an urban environment in this study [14,26]. The current study aimed (1) to examine the characteristics of bryophyte communities, and then to choose the most suitable indicator species in a large city (i.e., Wuhan) in central China, and (2) to assess the atmospheric environment in urban areas by using the bryophytes.

## 2. Materials and Methods

### 2.1. Study Area and Sampling

The study was conducted in the urban areas of Wuhan City, Hubei Province, China. Wuhan is a metropolis located at latitude 29° 58′–31° 22′ N and longitude 113° 41′–115° 05′ E. It is recognized as the main political, economic, financial, cultural, educational, and major transportation hub in central China, with a large population of 11.08 million. Abundant water bodies are distributed in the city and cover 26.1% of the city areas. Elevations of most city areas are below 50 m. The climate is characterized as subtropical, with distinct seasonal divisions, annual mean air temperature of 15.9 °C, and annual precipitation from 1100 mm to 1300 mm. As a major transportation hub, dozens of railways, roads, and expressways pass through Wuhan. Heavy industries, including steel works, chemical plants, and power plants, are also under development. Thus, the urban area of this city has mainly faced pollution from transportation and industry in recent decades.

**Figure 1:**
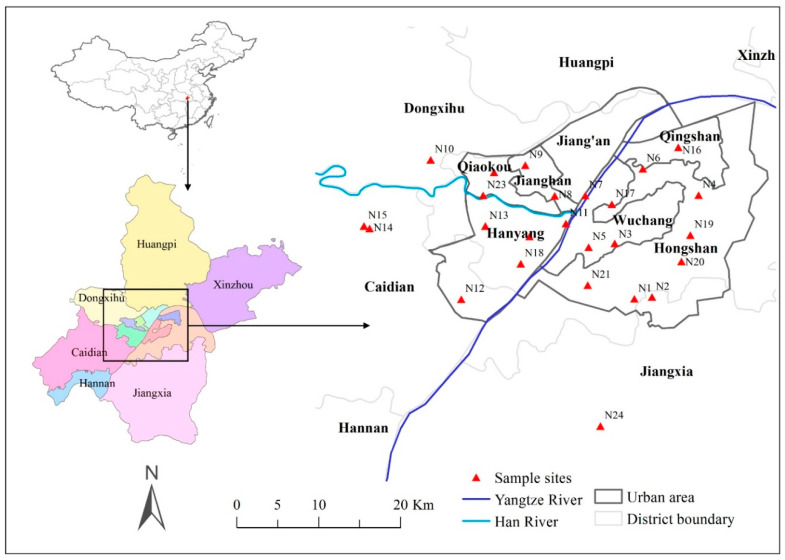
Map of the study area with sampling sites in the urban areas of Wuhan. N1–N25 are the sample sites, and detailed information is presented in Table S1.

Thirteen administrative divisions are included in Wuhan, of which seven divisions (i.e., Hanyang, Hongshan, Jiang’an, Jianghan, Qiaokou, Qingshan, and Wuchang) are considered urban areas (Figure 1). Twenty-five sites located within or nearby industry, transportation, university campus and residential areas were sampled on sunny days from September 2017 to November 2017. Detailed information of the 25 sampling sites, including locations and habitat environments, is displayed in Figure 1 and Table S1. At each sampling site, three 10 × 10 m sample plots were set, and then three to five 1 × 1 m were sampled in each sample plot where ground bryophytes occurred. These quadrats were at least 3 m away from the nearest tree in order to avoid the effects of the tree canopy. In each sample plot, trees with a diameter at breast height of more than 15 cm were investigated for epiphytic bryophytes, and three 10 × 10 cm quadrats at heights of 50, 80, and 130 cm for each tree were sampled from the single aspect with most bryophytes growing. The 1 × 1 m and 10 × 10 cm quadrats were divided into 100 equal squares to accurately measure the total coverage of the herb layer and the cover of individual bryophyte species. Coverage of tree canopy was calculated by visual estimation. The geographical location (longitude and latitude), altitude and the distance to the nearest roads of all sampling plots were also recorded. Bryophyte samples were collected by species for further classification and analyses, and underlying topsoil at a depth of 0–5 cm was also collected.

Bryophyte species were identified according to *Flora Bryophytarum Sinicorum* Vol.2–8 [27,28,29,30,31,32,33] and *Flora Yunnanica* Vol. 17 [34] in the laboratory. All specimens were stored at Huazhong Agricultural University. Soil samples for determining the soil water content were weighed before and after they were oven-dried at 105 °C to a constant weight. Samples for further chemical analyses were stored in plastic bags to avoid manual contamination.

### 2.2. Sample Preparation and Chemical Analysis

Moss samples (*Haplocladium angustifolium,* a dominant species with widespread distribution and high abundance) and soil samples were prepared for trace element analyses. Bryophyte samples (dominant species with widespread distribution and high abundance) were prepared by manually removing soil particles, dead materials, and litters. The green or greenish-brown parts of the bryophytes from dust particles were cleaned with deionized water. The bryophyte and soil samples were dried to a constant weight in a thermostatic drying machine for 48 h at 40 °C. The bryophytes were then ground to acquire fine powder in a mill, and the soil samples were homogenized with a mortar and pestle after coarse materials were removed using a 2 mm sieve. Three replicate measurements per bryophyte and soil sample were kept in clean, dry paper bags for further analyses.

Approximately 0.5 g of each bryophyte sample was transferred into a digestion tube and cold digested with 10 mL of mixed acid (HNO_3_:H_2_O_2_ = 4:1), and 0.25 g of each soil sample was digested with 10 mL of mixed acid (HNO_3_:HCl:HF = 3:1:1) for 30 min and then moved to a microwave oven (Mars 6, CEM, Matthews, NC, USA) for enhanced digestion until transparent solutions were obtained. After cooling, the digests were transferred to a 50 mL volumetric flask. The bryophyte was then filled with deionized water to 25 mL and soil to 50 mL. The presence and concentrations of trace elements were determined by inductively coupled plasma mass spectrometry (ICP-MS, Flexar LC-NexION 350X, PerkinElmer, Shelton CT, USA). The concentration of each element was corrected by subtracting blank values. A blank and a plant standard GBW07603 (GSW-2, IGGE, Langfang, China) or a soil standard GBW07403 (GSS-3, IGGE, Langfang, China) were analyzed to check the accuracy and precision of each element analysis. The recovery percentages of elements were >85% for quantitative analysis. All the detailed procedures of samples preparation and chemical analyses were referred to our previous study [35].

### 2.3. Data Analyses

#### 2.3.1. Diversity and Ordination Analyses for Bryophyte Species

The dominance of species in the study area was decided by the importance value, and a high importance value denotes the dominance of a species.

Importance value = (relative cover + relative frequency)/2,(1)

where frequency is calculated according to (sites of with bryophytes / total investigated sites).

Bryophyte α-diversity, characterized by Shannon–Wiener index, was calculated by the following equations:

(2) $$\mathrm{Shannon}–\mathrm{Wiener}\mathrm{index}:\;H=-\sum_{i=1}^{S}\left(P_{i}{\mathrm{lnP}}_{i}\right),$$

where S is the total number of species, that is, the species richness recorded at a specific sampling site. P_i_ = N_i_/N, where N_i_ is the relative cover of species i, and N is the sum of the relative covers of S species. 

Species distribution and environmental factors (soil water content, coverage of tree canopy and herb layer, habitat type, distance to the nearest roads, altitude) relationships were characterized by canonical correspondence analysis (CCA). Suitable biomonitoring species for tracing atmospheric trace elements were identified in this study on the basis of having high cover, frequency and importance value, and being widely distributed as recognized by CCA. CCA and the corresponding 2-dimensional ordination graphs were implemented in software CANOCO for Windows 4.5 (Microcomputer Power, Ithaca, NY, USA).

#### 2.3.2. Atmospheric Environment Assessment

The IAP [36] based on bryophyte species richness, cover, and frequency is an important approach for assessing atmospheric environments and is measured using the following equation:

(3) $$\mathrm{IAP}=\sum_{i=1}^{s}\left(Q_{i}\times f_{i}\right),$$

where s is the species richness at each sampling site; Q is an ecological index, which refers to the mean species richness of all sampled sites; and f is a comprehensive value of the cover and frequency of each bryophyte species, which was determined according to Gao and Cao [37].

The potential ecological risk index (RI), which reflects the potential ecological harm from a single metal and considers the integrated ecological effect of multiple elements [23,38], is another proposal for air quality assessment. The RI of multiple elements in a bryophyte sample was determined by

(4) $$\mathrm{RI}=\sum^{\;}E_{r}^{i}=\sum_{i}^{m}\left(T_{r}^{i}\times C_{f}^{i}\right)\;,$$

where $E_{r}^{i}$ presents the potential ecological risk coefficient of element i; m is the number of elements analyzed in the sample (m = 10 in the present study); $T_{r}^{i}$ is the toxic coefficient of a certain element; and $C_{f}^{i}$ is the contamination coefficient of element i, that is, $C_{f}^{i}=C^{i}/C_{n}^{i}$, where $C^{i}\;$is the measured concentration of element i, and $C_{n}^{i}$ is the background value of element i. The toxic coefficients of the 10 elements (i.e., As, Cd, Co, Cr, Cu, Mn, Ni, Pb, V, and Zn) were 10, 30, 5, 2, 5, 1, 5, 5, 2, and 1, respectively [38,39]. The concentration of the corresponding element in moss sample from a clean site remote from urban Wuhan was considered to be the background level ($C_{n}^{i}$) [40]. The classification criteria for potential ecological risk level are shown in Table S2 [38]. Five levels were established: (I) low risk, RI < 150; (II) moderate risk, 150 ≤ RI < 300; (III) considerable risk, 300 ≤ RI < 600; (IV) high risk, 600 ≤ RI < 1200; and (V) extreme risk, RI ≥ 1200.

#### 2.3.3. Statistical Analyses

The concentration values of trace elements from bryophyte and soil samples were given as minimum, maximum, mean, standard deviation (SD), and coefficient of variation (CV) for the 25 sampling sites. The statistical differences of each element concentration between the bryophyte and soil samples were determined by a paired-samples *t* test. The intercorrelations among site-specific elements, IAP and RI, were characterized by Pearson’s correlation analysis. The Shapiro–Wilk test of normality was conducted before parametric statistical analyses; the non-normal distribution dataset was transformed by log transformation.

## 3. Results

### 3.1. Diversity and Distribution of Bryophytes in the Urban Areas of Wuhan

At 25 sampling sites, 90 bryophyte species in 34 genera from 19 families were surveyed, with 67 species found on the ground and 44 species noted on the trunk of 61 trees. The families with the highest number of species present were Pottiaceae and Bryaceae, with almost one third of all surveyed species richness (Table S1). The species of the two families are small in size and have stems for erect growth forms, with a biomass relatively lower than that of creeping mosses. 

**Figure 2:**
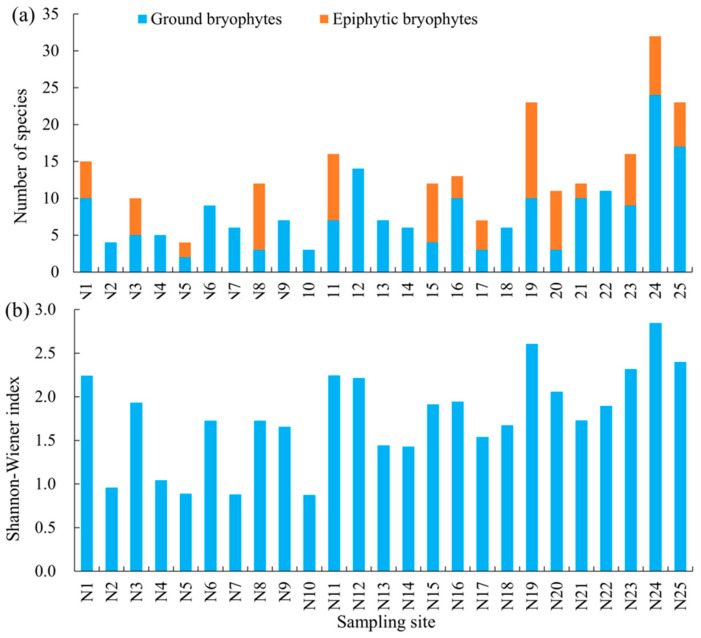
Bryophyte diversity of 25 sampling sites in urban areas of Wuhan: (**a**) number of species surveyed that grow on the ground or tree trunks and (**b**) α diversity index.

Within the 25 sampling sites, no epiphytic bryophytes species were found in 11 sites, such as N2, N4, N9, and N10. By contrast, N19, N24, and N25 sites showed a larger number of ground and epiphytic bryophyte species than the other sites (Figure 2a). Eight sampling sites (N1, N11, N12, N19, N20, N23, N24, and N25) had a Shannon–Wiener index higher than 2 (Figure 2b).

**Figure 3:**
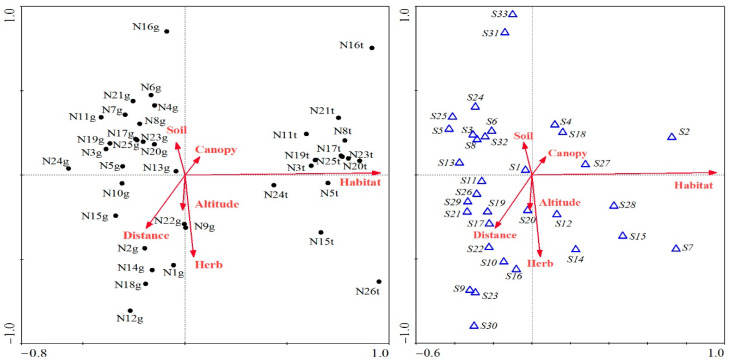
Canonical correspondence analysis of the relationships between bryophytes and environmental factors in Wuhan. The black points represent all sample sites with ground and epiphytic bryophytes, g means sample sites that showed ground bryophyte presence, and t indicates sample sites comprising epiphytic bryophytes. The blue triangles are the surveyed dominant bryophyte species, and the corresponding names are indicated in Table 2. Arrows are the environmental factors, where Habitat: habitat type of ground versus tree trunk; Canopy: coverage of tree canopy; Herb: coverage of herb layer; Distance: the distance to the nearest roads; Soil: soil water content; and Altitude: altitude of the sample sites.

The most dominant species with the highest importance value was *H. angustifolium*, which is a member of Thuidiaceae. This species has creeping main stems and pinnate branching systems, and the coverage and frequency were much higher than those of other species in the urban areas of Wuhan (Table 1). *H. angustifolium* (S1) seemed to have no preference and was distributed in all sampling sites, on the ground, and tree trunk (Figure 3). However, the epiphytic *H. angustifolium* was only found in six out of 14 sites. We selected *H. angustifolium* as a potential biomonitoring species in Wuhan and collected ground samples for elemental analyses. 

### 3.2. Trace Elements Present in H. angustifolium and Underlying Soil 

Trace elements in the samples of *H. angustifolium* and substrate soil were analyzed, as shown in Table 2. The element concentrations in *H. angustifolium* varied with the sampling sites, and the minimum and maximum values considerably differed (e.g., Co ranged from 1.27 mg kg^−1^ to 69.4 mg kg^−1^, and the coefficient of variation was even higher than 100%). The mean values of the trace element concentrations were in the following order: Mn > Zn > V > Cu > Cr > Pb > Ni > Co > As > Cd, with the Mn and Zn concentrations higher than 200 mg kg^−1^ and Cd levels lower than 1 mg kg^−1^. Although the concentrations of As and Cd were much lower than Mn and Zn, their contamination coefficients were much higher (10 and 30, respectively, versus 1); thus, they may be in a higher risk level. Comparatively, the concentrations of Cd and Zn in moss samples were significantly higher than those in soil samples (*p* < 0.01), whereas those of As, Cr, Mn, Pb, Ni, and V were significantly lower (*p* < 0.01). Among the 10 elements, the concentrations of As, Cd, Cu, Pb, and V in moss were significantly correlated with those in the substrate soil (*r* > 0.4, *p* < 0.05).

The intercorrelations of elements in *H. angustifolium* are presented in Table 3. As was highly significantly correlated with Co, Cr, and V; Co was highly significantly correlated with Cr, Mn, Ni, and V; Cr was highly significantly correlated with Mn and V; Mn was highly significantly correlated Ni; the correlation coefficients were higher than 0.7 *(p* < 0.01). Cd was significantly correlated with Pb and Zn; Cu was significantly correlated with Mn, Ni, Pb, V and Zn; Ni was significantly correlated with V; Pb was significantly correlated with Zn; the coefficient were higher than 0.44 (*p* < 0.05).

### 3.3. Assessment of Atmospheric Quality of the Urban Areas of Wuhan by IAP and RI through Bryophytes

Both IAP and RI were considered to assess the atmospheric environmental quality of urban Wuhan. As shown in Table 4, the mean values of $E_{r}^{i}$ for the trace elements were ranked as follows: Cd > As > Co > Cu > Ni > Pb > Cr > V > Mn > Zn. Cd had the highest mean value of $E_{r}^{i}$ (135.39), indicating considerable ecological risk, and contributed 34.5% to RI among all the elements. Additionally, As was also in considerable risk, while Co was in moderate ecological risk with an $E_{r}^{i}$ higher than 40. The study region was generally under considerable ecological risk, with the RI range of 93.77~831.87, and an average RI value of 392.83, and 56.0% of the study sites belonging to the considerable ecological risk level (300 < RI ≤ 600). 28% of the study area belonged to the moderate ecological risk level (150 < RI ≤ 300), 12.0% of the study sites belonged to the high ecological risk level (600 < RI ≤ 1200), and only one site (N24) was under a low risk (RI ≤ 150).

**Figure 4:**
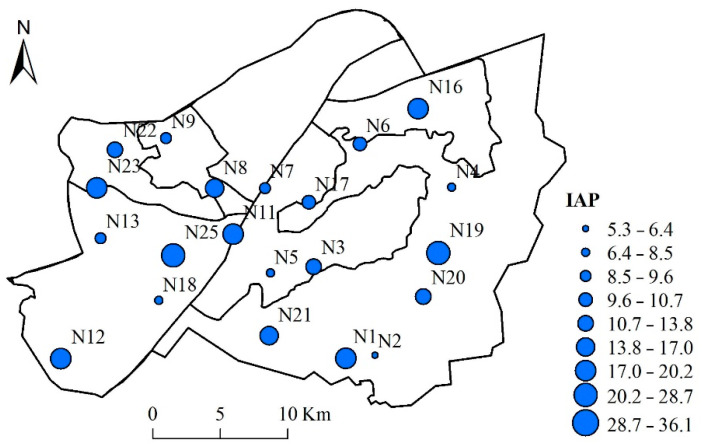
Spatial distribution of the index of atmospheric purity (IAP).

The appearance of IAP was somewhat in accordance with the spatial variation of RI. Sampling sites N15, N16, N19, N24 and N25 were found to be under low or moderate ecological risk, but they showed high IAP values, higher than 19 (Figure 4), suggesting a significant negative relationship between RI and IAP, with a correlation coefficient of −0.43 (*p* < 0.05; Table 3). Negative correlations were also found between IAP and trace elements, such as As, Co, Cr, Mn, and V (*r* < −0.4, *p* < 0.05; Table 3).

## 4. Discussion

### 4.1. Suitable Bryophyte Species as Biomonitors in Central China 

Terrestrial bryophytes have been widely used to monitor atmospheric depositions [12,41]. In this study, we suggested the combined usage of natural growing epigeic and epiphytic bryophytes to assess urban atmospheric environment. In addition to the bryophyte diversity in view of floristic composition and the presence and abundance of rare and significant taxa, the chemical analyses of certain bryophyte samples were also considered.

The major criteria for selecting a suitable species appear to be its widespread distribution and large abundance in the study region to ensure the availability of adequate material for capturing contaminants and chemical analyses for multiple sites [13,42]. However, a single unique species that is suitable for the biomonitoring of toxic element pollution worldwide has not been found yet [5]. Different moss species are used as biomonitors in different parts of the world. *Hylocomium splendens*, *H. cupressiforme*, and *P. schreberi*, particularly abundant in European countries, such as Italy, Kosovo, and Poland [10,17,43], have been most commonly used. *H. angustifolium*, a pleurocarpous feather moss, which demonstrates epigeic and epiphytic inhabitation in the study region, was used to evaluate the atmospheric environment in our study. This species is widely distributed in subtropical humid climate to warm temperate and semi-humid regions (from southern to central China) [44] and has been used for assessing atmospheric quality in several cities in China such as Guiyang, Shanghai, Wuhan, Wuxi, Xuzhou, and Taizhou [23,25,45].

### 4.2. Variations in Trace Element Accumulation in Moss Species

Mosses appear to be good biomonitors of atmospheric trace elements, such as Cd, Pb, Cu, V, and partially Zn [7]. In particular, the concentrations of Cd and Zn in moss species in our study region were significantly higher than those in soil, suggesting that *H. angustifolium* is a good biomonitor of certain elements. Mosses can be excellent biomonitors for certain elements, such as Cd or Pb, probably because these elements almost exclusively originate from the atmosphere [9]. The most important source of Cd and Pb in an urban environment is road transportation [46]. Pollution from industrial emissions is the main source of metal pollution in China [47]. In Wuhan, anthropogenic activities have an important impact on the accumulation of trace elements in soil; vehicular emissions, industrial activities, and household waste may be the three main sources of accumulated trace elements, and Cd may be the largest accumulated trace element in soil and soil pollution factor [48]. As also demonstrated by Gong et al. [47] in urban–rural topsoil in Wuhan, Cd, Cu, Hg, Pb, and Zn were mostly derived from anthropogenic inputs; Co, Cr, and Mn were controlled by natural sources; and Ni seemed to be affected by anthropogenic and natural sources. 

Soil may be another source of trace elements accumulated in mosses. Although mosses do not take up substances directly from soil, local soil particles may strongly influence the chemical composition in mosses by blowing wind; the entrapped particles on the moss surfaces are then washed down and partly dissolved by precipitation, thereby enriching several elements in mosses [42,49]. Other factors, such as topography, rainfall, wind direction, and vegetation conditions, often influence the concentration of trace elements in mosses [50], thereby resulting in a spatial divergence in trace element distribution. For example, in two closed university sites, N1 and N2, in a South Lake neighborhood, differences were observed in bryophyte species richness and concentrations of most trace elements (e.g., Cd, Co, Cr, Mn, Ni, V, and Zn), probably in relation to the differences of microtopography, soil properties and vegetation conditions. 

Several trace elements in moss samples showed significant correlations. For example, positive correlations between the concentrations of Cd and Pb in mosses were found in this study and in most European countries [10,51]; this finding was in agreement with the results of several investigations in other cities in China, such as Taizhou and Xuzhou [23,52]. These results suggested that these trace elements often appear simultaneously and may come from the same source of pollution. 

### 4.3. Using IAP and RI to Assess Environmental Quality by Bryophytes

IAP, which was introduced by le Blanc and de Sloover [36], is one of the most popular indicators of atmospheric environments [14,53]. IAP was analyzed in terms of species richness, cover, and frequency, and it was highly consistent with the bryophyte α-diversity of the Shannon–Wiener index in the study. For instance, N1, N11, N12, N19, N23, N24, and N25 presented high values of IAP and Shannon–Wiener index. Bryophyte occurrence and coverage were significantly influenced by tree properties and vegetation type of the site [26]. Land use intensity, land cover types, and disturbance also affect bryophyte diversity through several different mechanisms, such as light levels and environmental heterogeneity [54]. The aforementioned sampling sites in the study area either possess a relatively high vegetation cover and/or with green hills and have a certain distance to the main road that reduces anthropogenic disturbances, thereby allowing the maintenance of abundant bryophytes and high IAP. Air pollution usually negatively influences the occurrence of sensitive bryophyte species and decreases IAP [26]. Not surprisingly, negative but not very strong correlations were found for IAP and several trace elements in this study. 

Generally, RI was significantly negatively correlated with IAP for the study sites, the spatial variation in RI was somehow negatively in accordance with IAP. Unlike the diversity index IAP, RI evaluates the contamination levels of several trace elements’ composition in biomonitors. For the RI of soil trace elements, approximately 72.4% of the urban areas in Wuhan have reached the level of considerable potential ecological risk (300 ≤ RI < 600) [48]. Similarly, 56.0% of our study sites were assessed as being in the considerable potential ecological risk level. This ecological risk level was also comparable with that in Taizhou, a city in the east of China with heavy industrial activities [23]. As mentioned above, Cd was recognized as the largest soil pollution factor in Wuhan [48], and it was also strongly accumulated in moss and contributed highest to the integrated RI (29.4%). Therefore, Cd is a high-risk element that requires further attention. Apart from Cd, other trace elements, such as As and Co, also contributed a great deal to RI, thus requiring caution.

## 5. Conclusions 

The use of the diversity of bryophytes and the accumulated trace elements in them in urban areas of Wuhan City exhibited a clear spatial pattern of bryophyte functional richness and the concentrations of 10 elements (As, Cd, Co, Cr, Cu, Mn, Ni, Pb, V, and Zn). Environmental heterogeneity greatly influenced the occurrence and abundance of bryophytes in terms of varied life forms, coverage, and IAP. Among all of the species in the urban areas, *H. angustifolium*, which is a pleurocarpous feather moss, appeared to be a good biomonitor of atmospheric quality in central China. The urban areas in Wuhan generally faced a considerable potential ecological risk level caused by atmospheric trace element pollution, and Cd exhibited the highest ecological risk level among the 10 elements. This study suggests that effective measures should be taken into consideration to alleviate certain trace element contamination in the atmosphere and reduce the ecological risk level of trace elements in this major metropolis.

## Figures and Tables

**Table 1:** Dominant bryophyte species and their important values.

No.	Species	Genus	Family	Coverage (%)	Frequency (%)	Important Value
S1	*Haplocladium angustifolium*	*Haplocladium*	Thuidiaceae	15.56	77	0.140
S2	*Entodon obtusatus*	*Entodon*	Entodontaceae	7.75	41	0.071
S3	*Fabronia curvirostris*	*Fabronia*	Fabroniaceae	4.45	15	0.035
S4	*Claopodium aciculums*	*Claopodium*	Thuidiaceae	3.52	21	0.034
S5	*Plagiomnium cuspidatum*	*Plagiomnium*	Mniaceae	4.31	13	0.033
S6	*Weissia exserta*	*Weissia*	Pottiaceae	1.25	26	0.025
S7	*Schwetschkeopsis fabronia*	*Schwetschkeopsis*	Fabroniaceae	3.10	10	0.024
S8	*Sematophyllum phoeniceum*	*Sematophyllum*	Sematophyllaceae	2.11	15	0.022
S9	*Fissidens adelphinus*	*Fissidens*	Fissidentaceae	1.82	15	0.021
S10	*Bryum coronatum*	*Bryum*	Bryaceae	1.44	18	0.020
S11	*Bryum dichotomum*	*Bryum*	Bryaceae	1.59	15	0.019
S12	*Physcomitrium sphaericum*	*Physcomitrium*	Funariaceae	1.58	15	0.019
S13	*Taxiphyllum taxirameum*	*Taxiphyllum*	Hypnaceae	0.86	21	0.019
S14	*Bryum argenteum*	*Bryum*	Bryaceae	0.75	21	0.018
S15	*Hyophila stenophylla*	*Hyophila*	Pottiaceae	1.69	13	0.018
S16	*Barbula subcontorta*	*Barbula*	Pottiaceae	0.87	18	0.017
S17	*Weissia microstoma*	*Weissia*	Pottiaceae	0.85	18	0.017
S18	*Claopodium rugulosifolium*	*Claopodium*	Thuidiaceae	1.15	15	0.017
S19	*Taxiphyllum subarcuatum*	*Taxiphyllum*	Hypnaceae	1.09	15	0.017
S20	*Taxiphyllum cusoidifolium*	*Taxiphyllum*	Hypnaceae	1.68	10	0.016
S21	*Brachythecium amnicolum*	*Brachythecium*	Brachytheciaceae	1.62	10	0.016
S22	*Fissidens bryoides*	*Fissidens*	Fissidentaceae	0.83	15	0.015
S23	*Brachythecium plumosum*	*Brachythecium*	Brachytheciaceae	1.94	5	0.014
S24	*Venturiella sinensis*	*Venturiella*	Erpodiaceae	1.46	8	0.013
S25	*Brotherella nictans*	*Brotherella*	Sematophyllaceae	1.29	8	0.012
S26	*Weissia controversa*	*Weissia*	Pottiaceae	0.62	13	0.012
S27	*Entodon plicatus*	*Entodon*	Entodontaceae	1.13	8	0.012
S28	*Entodon scariosus*	*Entodon*	Entodontaceae	1.06	8	0.011
S29	*Brotherella fauriei*	*Brotherella*	Sematophyllaceae	1.05	8	0.011
S30	*Oxystegus cuspidatus*	*Oxystegus*	Pottiaceae	1.62	3	0.011
S31	*Eurhynchium laxirete*	*Eurhynchium*	Brachytheciaceae	0.99	8	0.011
S32	*Fabronia matsumurae*	*Fabronia*	Fabroniaceae	1.08	5	0.010
S33	*Frullania parvistipula*	*Frullania*	Frullaniaceae	1.39	3	0.010

**Table 2:** Trace element concentrations (mg kg^-1^) in moss *Haplocladium angustifolium* and the substrate soil.

Material	Metal	As	Cd	Co	Cr	Cu	Mn	Ni	Pb	V	Zn
Moss	Maximum	19.5	1.68	69.3	72.5	77.7	704	50.8	47.0	96.3	458.0
	Minimum	1.33	0.276	1.27	5.39	9.31	71.5	5.00	8.03	10.0	48.7
	Mean	8.21	0.776	14.4	33.8	36.7	348	19.0	25.11	38.9	214
	SD	4.78	0.32	16.6	16.8	17.1	178	12.0	11.6	21.7	105
	CV (%)	58.3	41.3	115	49.8	46.4	51.0	63.5	46.1	55.8	48.9
Soil	Maximum	34.3	0.737	22.7	144	80.5	952	51.8	67.5	219	170.5
	Minimum	13.3	0.11	6.47	55.5	24.9	163	15.4	13.8	41.3	41.6
	Mean	21.1	0.353	15.2	83.6	38.8	693	31.8	38.1	127	82.5
	SD	5.24	0.185	3.69	20.6	12.7	190	8.64	16.2	45.7	34.9
	CV (%)	24.8	52.2	24.3	24.6	32.8	27.5	27.2	42.6	35.8	42.3
Difference (*P*)		0.000	0.000	0.016	0.000	0.293	0.000	0.000	0.003	0.000	0.000
Pearson’s correlation		0.458 *	0.416 *	0.303	0.387	0.479 *	0.268	0.176	0.414 *	0.512 **	0.272

Note: SD: standard deviation; CV: coefficient of variation; Difference (*P*) shows the results of paired- samples *t* test between the concentrations of elements in soil and moss samples, where *p* < 0.05 means a significant difference, and *P* < 0.01 means a highly significant difference; * Correlation is significant at the 0.05 level. ** Correlation is significant at the 0.01 level.

**Table 3:** Correlations of trace elements in *Haplocladium angustifolium*, the index of atmospheric purity (IAP), and potential ecological risk index (RI).

	As	Cd	Co	Cr	Cu	Mn	Ni	Pb	V	Zn	IAP
Cd	0.29										
Co	0.76 **	0.12									
Cr	0.72 **	−0.01	0.77 **								
Cu	0.60 **	0.33	0.61 **	0.62 **							
Mn	0.48 *	−0.04	0.86 **	0.70 **	0.49 *						
Ni	0.35	0.07	0.79 **	0.60 **	0.44 *	0.90 **					
Pb	0.48 *	0.54 *	0.43 *	0.27	0.52 *	0.34	0.34				
V	0.75 **	−0.08	0.75 **	0.89 **	0.44 *	0.68 **	0.54 *	0.24			
Zn	0.06	0.44 *	0.09	0.08	0.44 *	0.10	0.15	0.47 *	−0.15		
IAP	−0.54 *	0.03	−0.5 *	−0.58 **	−0.39	−0.47 *	−0.28	−0.27	−0.59 **	−0.01	
RI	0.86 **	0.49	0.87 **	0.71 **	0.64 **	0.69 **	0.65 **	0.60 **	0.67 **	0.25	−0.43 *

* Correlation is significant at the 0.05 level. ** Correlation is significant at the 0.01 level.

**Table 4:** Potential ecological risk assessment of atmospherically deposited trace elements by mosses in Wuhan. $E_{r}^{i}:$ the potential ecological risk coefficient of element i; RI: potential ecological risk index of multiple elements.

Sample Site	$\mathrm{Potential}\;\mathrm{Ecological}\;\mathrm{Risk}\;\mathrm{of}\;\mathrm{Element}\;i\;(E_{r}^{i})$										RI	Category
	As	Cd	Co	Cr	Cu	Mn	Ni	Pb	V	Zn		
N1	95.71	164.65	8.66	4.46	13.02	1.35	11.21	12.11	3.94	8.88	324.00	III
N2	116.06	94.71	26.87	11.14	12.91	3.14	21.48	12.37	10.35	3.49	312.52	III
N3	105.52	145.64	22.80	13.77	22.62	2.88	21.58	11.39	10.85	4.31	361.34	III
N4	72.43	140.58	39.26	9.16	15.92	2.75	19.68	9.88	8.55	3.74	321.94	III
N5	97.34	122.97	44.68	15.97	40.85	4.62	25.57	27.64	10.75	7.49	397.88	III
N6	97.52	138.31	55.02	14.08	19.53	3.35	22.31	14.72	9.08	6.95	380.87	III
N7	181.39	192.91	99.72	20.38	31.98	5.00	37.64	23.65	15.26	6.39	614.31	IV
N8	136.44	144.77	90.12	16.76	34.55	4.11	33.41	14.28	14.24	7.53	496.22	III
N9	130.95	130.99	183.10	18.43	16.70	7.13	41.19	12.12	16.93	4.02	561.54	III
N10	197.26	147.21	312.77	27.11	22.80	8.43	64.44	24.58	23.39	3.89	831.87	IV
N11	138.23	212.27	191.49	10.90	24.54	5.32	76.41	25.33	6.40	6.68	697.55	IV
N12	58.58	75.17	111.48	11.76	23.28	7.29	85.29	12.03	7.88	6.79	399.55	III
N13	97.43	94.36	146.25	26.06	30.83	8.74	102.34	10.90	16.61	11.81	545.33	III
N14	95.98	87.38	90.07	17.50	23.96	8.20	73.60	9.69	13.35	3.87	423.59	III
N15	62.47	91.22	17.70	18.01	9.81	2.66	34.92	9.77	14.62	2.80	263.98	II
N16	39.44	118.60	24.08	8.07	12.11	4.53	36.88	12.44	11.46	2.95	270.56	II
N17	26.83	143.90	13.91	4.84	12.52	3.01	25.60	21.28	3.66	12.63	268.17	II
N18	39.50	88.43	23.05	9.18	11.73	4.09	31.63	9.44	7.28	3.23	227.57	II
N19	39.76	118.95	17.16	7.57	9.86	2.59	26.42	14.37	4.78	5.94	247.41	II
N20	37.69	156.63	29.95	7.66	15.74	6.00	55.90	20.94	5.94	4.32	340.77	III
N21	76.28	292.33	23.07	13.01	30.07	3.71	31.56	27.88	5.46	11.04	514.39	III
N22	28.86	57.21	12.57	12.28	15.60	2.02	17.33	5.67	3.31	4.42	159.26	II
N23	43.40	242.09	17.55	7.14	15.30	2.64	28.30	6.55	4.48	6.09	373.54	III
N24	13.48	48.14	5.73	2.01	4.89	0.89	10.09	4.76	2.43	1.34	93.77	I
N25	47.14	132.91	19.93	9.07	11.99	3.56	21.18	18.54	5.43	7.18	276.93	II
Average	84.52	135.39	66.96	12.80	19.63	4.35	38.95	14.74	9.62	5.86	392.83	III

## Supplementary Materials

The following are available online at https://www.mdpi.com/1660-4601/17/12/4537/s1, Table S1: Description of sample sites in urban areas of Wuhan, China, Table S2: Classification standards of potential ecological risk indexes (RI), Table S3: Families with genus and species richness of bryophytes in urban areas of Wuhan.
