# Supplementary material for: Urban Atmospheric Environment Quality Assessment by Naturally Growing Bryophytes in Central China
Source: Int J Environ Res Public Health. 2020 Jun 24;17(12):4537. doi: 10.3390/ijerph17124537 (PMC7344691; doi:10.3390/ijerph17124537)
Supplement: Supplementary file 1 [file ijerph-17-04537-s001.pdf]

Supplementary Materials

Table S1. Description of sample sites in urban area of Wuhan, China.

Sample Site	Site Name	Eco-functional Region	Latitude/ E (°)	Longitude/ N (°)	Coverage of Trees (%)	Coverage of Herbs (%)	Soil Water Content (%)	Distance to Main Roads
N1	Huzhong Agricultural University	University campus	114.357725	30.4727305	36	48	23.8	780
N2	Zhongnan University of Economics and Law	University campus, transportation	114.377167	30.4746917	44	39	15.8	920
N3	Shiyang Martyrs Cemetery	Scenic pot	114.336225	30.5336	60	12	6.0	80
N4	City College, Wuhan University of Science and Technology	University campus	114.428236	30.5865777	37	22	9.2	270
N5	Ziyang Park	Transportation	114.307414	30.5295083	52	15	5.9	400
N6	Garden Science Park	Scenic pot	114.366922	30.6155361	45	16	10.1	100
N7	Hankou Jiangtan	Transportation	114.303783	30.5860444	58	13	21.4	100
N8	Zhongshan Park	Scenic pot	114.270222	30.5858806	49	13	25.7	400
N9	Changqing	Residential	114.2378472	30.6192666	46	34	25.0	800
N10	Wujiashan	Industry	114.1341139	30.6252639	64	7	17.3	60
N11	Guishan Scenic Area	Transportation	114.2824166	30.5554222	56	4	13.5	40
N12	Tanghu Park	Industry	114.167655	30.4724083	42	23	15.2	1500
N13	Longyang Lake Park	Industry	114.1941166	30.5526861	80	10	19.2	1200
N14	Houguan Lake Wetland Park	Scenic pot	114.066955	30.5499139	30	12	19.1	1200
N15	Zhushan Lake Park	Industry	114.0607972	30.5527	50	2	16	150
N16	Qingshan Park	Industry	114.4058861	30.6388889	39	15	24.8	300
N17	Hubei University	University campus	114.3329361	30.5765222	60	21	16.2	200
N18	Wuhan International Expo Center	Scenic pot	114.2329667	30.5113472	50	31	24.6	1600
N19	Wuhan Botanic Garden	Scenic pot	114.4193583	30.5427194	48	10	24.1	3000
N20	Huazhong University of Science and Technology	University campus	114.409375	30.5137028	41	32	30.4	700
N21	Wuchang Shouyi University	Transportation	114.3064361	30.4877861	35	12	25.5	200
N22	Hankou Spring	Residential	114.2036083	30.611525	41	32	24.2	160
N23	University of Military and Economics	University campus	114.1916694	30.5861972	32	7	32.3	600
N24	Qinglongshan National Forest Park	Scenic pot	114.3206	30.3332056	35	5	30.7	1600
N25	Wuhan Zoo	Scenic pot	114.242522	30.5411417	50	17	42.4	600

Table S2. Classification standards of potential ecological risk indexes (RI)

E_r^i	RI	Level	Category
< 40	< 150	low risk	I
40~80	150~300	moderate risk	II
80~160	300~600	considerable risk	III
160~320	600~1200	high risk	IV
≥ 320	≥ 1200	extremely risk	V

Table S3. Families with genus and species richness of bryophytes in Wuhan's urban areas.

Order	Family	Genus Richness	Percentage of Genus Richness (%)	Species Richness	Percentage of Species Richness (%)
1	Pottiaceae	5	14.3	13	14.4
2	Bryaceae	3	8.6	13	14.4
3	Entodontaceae	1	2.9	11	12.2
4	Brachytheciaceae	3	8.6	8	8.9
5	Sematophyllaceae	2	5.7	7	7.8
6	Hypnaceae	2	5.7	6	6.7
7	Thuidiaceae	2	5.7	5	5.6
8	Frullaniaceae	1	2.9	4	4.4
9	Fissidentaceae	1	2.9	3	3.3
10	Funariaceae	1	2.9	3	3.3
11	Fabroniaceae	2	5.7	3	3.3
12	Mniaceae	1	2.9	3	3.3
13	Leskeaceae	1	2.9	2	2.2
14	Geocalycaceae	1	2.9	2	2.2
15	Dicranaceae	2	5.7	2	2.2
16	Erpodiaceae	2	5.7	2	2.2
17	Polytrichaceae	1	2.9	1	1.1
18	Aytoniaceae	1	2.9	1	1.1
19	Amblystegiaceae	1	2.9	1	1.1
